# Differential risk factors for prenatal and postpartum depression in South Korea

**DOI:** 10.1192/j.eurpsy.2021.1599

**Published:** 2021-08-13

**Authors:** S.H. Park, K. Chung, H.Y. Cho, Y.R. Kim, K. Jhung

**Affiliations:** 1 #405 Biomedical Research Institute, Catholic Kwandong University International St. Mary’s Hospital, INCHEON METROPOLITAN CITY, Korea, Republic of; 2 Department Of Psychiatry And Institute Of Behavioral Science In Medicine, Yonsei University College of Medicine, Yonsei University Health System, Yongin, Korea, Republic of; 3 Department Of Obstetrics And Gynecology, CHA Gangnam Medical Center, CHA University, Seoul, Korea, Republic of; 4 Department Of Obstetrics And Gynecology, CHA bundang Medical center, CHA University, Seongnam-si, Korea, Republic of; 5 Department Of Psychiatry & Behavioral Neuroscience, Catholic Kwandong University International St. Mary’s HospitalCatholic Kwandong University International St. Mary’s Hospital, INCHEON Metropolitan city, Korea, Republic of

**Keywords:** Perinatal depression, postpartum period, Risk factors, gestation period

## Abstract

**Introduction:**

Incidence for depression increases during the perinatal period. Risk factors for depression may differentially affect each time period.

**Objectives:**

To assess demographic, psychological and obstetric risk factors that differentially affect prenatal and postpartum depression

**Methods:**

A total of 169 subjects participated. Assessment was conducted during the first trimester, second trimester, third trimester, within a month after childbirth, and a month after childbirth. Demographic and obstetric measures, as well as psychological measures, including the Edinburgh Postnatal Depression Scale were conducted. Multiple regression and the Mann-Whitney U test were performed to examine the association between variables and depression scores.

**Results:**

Depression score was higher during the postpartum period than the prenatal period. Younger age was associated with depression during the first trimester. In the second trimester, less education, a history of depression and having stress within a year significantly affected depression scores. Smoking, artificial abortion and lack of support from family and parents correlated with depression during the third trimester. Within a month after childbirth, psychiatric and depression history, smoking, stress level within a year and lack of family support were associated with depression. At a month after childbirth, those who were primiparous and not breastfeeding had significantly higher depression scores.

**Conclusions:**

This study identifies various risk factors for each gestational and postpartum period and suggests differential interventions for different perinatal periods.

